# Case Report: Targeted interleukin-6 blockade by siltuximab for cytokine release syndrome control and infection limitation in thirteen patients treated with bi-specific T-cell engagers

**DOI:** 10.3389/fimmu.2026.1749348

**Published:** 2026-02-12

**Authors:** Jean-François Rossi, Thierry Cailleux, Emma Wisnewski, Julie Coussirou, Françoise de Crozals

**Affiliations:** 1 Faculté de Médecine-Université de Montpellier, Montpellier, France; 2 Hematology Department, Institut du Cancer Avignon-Provence, Avignon, France

**Keywords:** anti-interleukin-6 therapy, bispecific T-cell engagers, infection, lymphoma, multiple myeloma

## Abstract

Infections remain a major concern during treatment with bispecific T-cell engagers (BiTE) in hematological malignancies. The risk is primarily driven by disease- and treatment-related immunosuppression, as well as corticosteroid use during the early phase of therapy. It persists throughout the treatment course, requiring appropriate prophylactic measures. We report a real-world series of thirteen patients treated with BiTEs: 10 with multiple myeloma, two with refractory AL amyloidosis, and one with mantle cell lymphoma. During initial hospitalization, daily C-Reactive protein (CRP) monitoring was performed. Siltuximab, an anti-interleukin-6 monoclonal antibody, was administered instead of corticosteroids when CRP exceeded 40 mg/L with a rapid 24-hour increase, based on predictive mathematical modeling, to preempt cytokine release syndrome (CRS). All patients received standard anti-infective prophylaxis, and treatment duration was adapted to clinical response. This approach was associated with only three grade 1–2 infections, indicating a favorable safety profile. These preliminary results support the design of a prospective multicenter study to evaluate the feasibility of home-based BiTE administration, integrating point-of-care testing, a digital health platform, and 24/7 remote monitoring via a dedicated call center, within a comprehensive medico-economic framework.

## Introduction

Immunotherapy represents a breakthrough in oncology, particularly for hematological malignancies. Among these, bi-specific T-cell engagers (BiTEs) have received approval for the treatment of relapsed or refractory B-cell acute lymphoblastic leukemia (ALL), multiple myeloma (MM), and various lymphomas, including diffuse large B-cell lymphoma (DLBCL), mantle cell lymphoma (MCL) and follicular lymphoma (FL). BiTEs have demonstrated high response rates in these settings and are now being considered for incorporation into frontline therapy. Notably, their use may eliminate the need for autologous stem cell transplantation as suggested in emerging MCL treatment strategies (1).

However, infectious complications remain a major concern. In phase II trials of teclistamab and elranatamab for MM, infections were reported in 76.4% and 69.9% of patients, respectively, with grade 3–4 infections in 44.8% and 39.8%, and infection-related mortality reaching 9.7% and 6.5%, respectively (2, 3). An analysis of the WHO VigiBase pharmacovigilance database by Contejean et al. (4) identified infections in 188 of 692 patients (25.4%) treated with B-cell maturation antigen (BCMA) BiTEs. Similarly, real-world data have shown infection rates in 56.2%, with 21.9% of patients experiencing grade ≥3 events, primarily bacterial pneumonias (5). These infections were closely associated with corticosteroid use, employed to manage cytokine release syndrome (CRS) and immune effector cell–associated neurotoxicity syndrome (ICANS) (6). The infection risk is intrinsically linked to both disease- and treatment-related immunosuppression, highest during the first two months of therapy, thus warranting prophylactic anti-infective strategies (6). These concerns emphasize the need to re-evaluate treatment duration and to develop personalized strategies guided by risk and response (7).

To safely implement BiTEs use in real-world settings, three critical elements are essential: (1) effective CRS management without corticosteroids to reduce infection risk, (2) appropriate prophylactic anti-infective measures and, (3) remote monitoring supported by point-of-care testing (POCT) and digital health tools. In a small real-world cohort, we applied these principles using anti-Interleukin-6 (IL-6) therapy, traditionally reserved for steroid-refractory CRS (3, 4). We opted for siltuximab, an anti-IL-6 monoclonal antibody (Sylvant®, Recordati Rare Diseases, Puteaux, France) guided by daily C-reactive protein (CRP) monitoring and mathematical modeling to enable early intervention (8). This strategy now forms the basis of a new prospective study integrating digital health monitoring, POCT and a dedicated 24/7 support platform to ensure safe and feasible home-based BiTE administration.

## Case presentation

All patients were informed about this “real-life” analysis. They received various BiTEs under authorization for standard indications and were included in the French Therapeutic Use and Data Collection Program (Programme d’Utilisation Thérapeutique et de Recueil de Données, PUT-RD), overseen by the French Drug Agency (Agence Nationale de Sécurité du Médicament et des Produits de Santé, ANSM).

Treatments included anti-CD3-anti-BCMA BiTEs, elranatamab (Elrexfio®, Pfizer, Paris, France), or teclistamab, (Tecvalyi®, Johnson & Johnson, Issy-les-Moulineaux, France), and glofitamab (Columvi®, Roche, Boulogne-Billancourt, France), an anti-CD3-anti-CD20 BiTE. Dosing and administration followed manufacturers’ protocols, with one key modification in premedication: 5 mg of IV dextropheniramine and 1000 mg of paracetamol, given 1–3 hours prior to the first dose. Corticosteroids were strictly avoided due to their immunosuppressive effect and associated increased risk of infection. Teclistamab was administered subcutaneously (sc), with step-up dosing: 0.06 mg/kg on day 1, 0.3 mg/kg on day 3 and 1.5 mg/kg on day 5, followed by weekly maintenance at 1.5 mg/kg. Elranatamab was also given sc,: 12 mg on day 1, 36 mg on day 4, and 76 mg weekly thereafter. Glofitamab was administered intravenously (IV) one week following obinutuzumab, beginning at 2.5 mg, increasing to 10 mg after five days, and then 30 mg every 21 days.

CRP levels were measured twice daily in the first two patients and once daily in subsequent patients during the first week, then weekly prior to each dose. Siltuximab was administered IV at the standard dose (11mg/kg), when CRP exceeded 40 mg/L with a rapid rise within 24hours, based on our mathematical modeling and data observed during CRS in COVID-19 and CAR T-cell therapy (1–3, 8). Following the third BiTE dose, all patients received either monthly intravenous immunoglobulins (IVIG) or weekly subcutaneous immunoglobulins, along with valaciclovir and trimethoprim–sulfamethoxazole for infection prophylaxis. Immunoglobulin replacement therapy was continued throughout the treatment period and prolonged in cases of severe hypogammaglobulinemia.

As shown in **Table 1**, a total of thirteen patients were treated: ten with MM, two with AL and one with MCL. Seven patients received elranatamab, five received teclistamab and one received glofitamab. All the patients were adults over 55 years-old having contraindications to the use of Chimeric Antigen Receptor T-cells. All patients had relapse or refractory disease, having received 3 to 6 prior lines of therapy. Eight patients received siltuximab either before or after the second dose of BiTE treatment. Only Patient 1 required two siltuximab doses due CRP re-increase after the second dose.

**Table 1:** Patient demographics, disease features, and treatment.

Pts	Stage R-ISS B2MG (mg/)L EMD*	Diagnosis BiTE MoAb	Line of Tt	Siltuximab	CRS	Infection	Response	Duration of Tt (Time without Tt)
1	2 4.5 EMD+	MM IgGk Elra	5	2 doses (D) Post-D1 and D3	No	0	dead in PD 6 mo.	4 mo.
2	2 4.41 EMD-	MM IgGk Elra	4	Post-D1	No	0	CR	4 mo. (8 mo.)
3	2 3.04 EMD-	MM IgAk Elra	4	Post-D1 1: 246.3	No	0	PR dead in PD at 8 mo	4 mo.
4	3 12.37 EMD+	MM IgAk Elra	6	Post-D1	No	0	Dead in PD	3 mo
5	3 9.29 EMD-	MM IgAk Elra	3	Post-D1	No	0	dead in PD	2 mo.
6	1 3.1 EMD-	MM IgG L Tecli	3	No	No	0	CR	4mo. (5mo.)
7	1 2.3 EMD-	MM IgGk Tecli	4	No	Gr 1	Bacterial Pneum. Gr 2	CR	4 mo (11 mo.)
8	2.97 LDH+ EMD+	lambda AL Tecli	3	No	no	0	CR	2 mo (12mo)
9	2 3.04 EMD-	MM k Tecli	3	No	No	Bacterial Pneum. Gr 2	PD	Dead PD (1 mo.)
10	1 3.2 EMD-	MM IgGk Elra	3	Post-D1	No	0	CR	3 mo. (6 mo.)
11	2 4.19 EMD+	MM IgAL Elra 1^st^ cycle 4 mo. 6mo No Tt 2^nd^ cycle 4 mo.	3	No (corticosteroid) Post-D1	Gr2 No	Pneum. Gr1	CR MRD + CR MRD -	4 mo. 4 mo. (6 mo.)
12	3.2 LDH+ EMD+	AL Lambda Tecli	3	No	Gr1	0	PR	4 mo (6 mo.)
13	IVBb*	MCL Glofitamab	6	Post-D1	No	0	CR	CR (10 mo.)

EMD, Extra-medullary disease; BMG, beta2 microglobulinemia (mg/L); R-ISS, revised International Staging system; *, IPI staging lymphoma; PD, Progressive disease; MM, Multiple myeloma; Elra, elratamab; Tecli, teclistamab; BiTE, Bispecific T-cell engager; CRS, Cytokine release syndrome; M, male; F, female; mo, months; Pneum, pneumonia; CR, Complete response; k, Kappa; L, lambda; D, day; MRD, minimal residual disease; Tt, treatment; Gr, grade; PR, partial response; AL, amyloidosis; MCL, mantle cell lymphoma.

**Figure 1:**
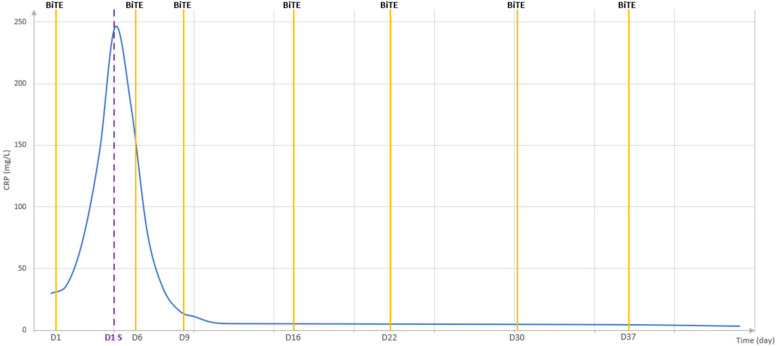
Dynamics of CRP in patient 3 following administration of siltuximab one day after the first injection of elranatamab. As shown, CRP levels increased rapidly, reaching a peak of 246.3 mg/L, and then declined promptly after siltuximab administration. This rapid decrease enabled the safe administration of subsequent elranatamab doses, with CRP levels returning to the normal range within the following five days.

As shown in **Figure 1**, CRP levels were rapidly controlled within 24 hours of siltuximab administration, consistent with the kinetics of CRP production in response to IL-6 and its half-life (approximately 18 hours). No CRS events were observed. Four patients did not receive siltuximab due to the absence of a CRP increase greater than 40mg/L within 24 hours, and one patient was excluded from siltuximab treatment due to a CRP rise associated with an active infection.

Infections were limited to three cases within three months of BiTE initiation: two grade 2 (pneumonia), and one grade 1. Treatment duration was personalized, particularly in elderly patients, based on clinical response. Eight patients achieved a complete response (CR). Minimal Residual Disease (MRD) was assessed by flow cytometry in three, all of whom were negative, including one patient retreated with elranatanab after biological relapse (re-increase of free light chain serum level), again achieving MRD negativity.

## Discussion

The prognosis of B-cell malignancies has been significantly improved by the advent of immunotherapies, notably chimeric antigen receptor T-cells (CAR) and BiTEs (1). While both approaches demonstrate high efficacy, BiTEs, particularly in subcutaneous formulations, offer logistical advantages, enabling outpatient and potentially home-based administration. This is especially relevant for elderly patients, who are often excluded from CAR-T therapy.

Outpatient administration of BiTEs has been evaluated using remote monitoring kits that allow patients to track vital signs and remain connected to a central command center during the step-up dosing period to monitor CRS and ICANS. However, the initial step-up doses are typically administered in a hospital setting before transitioning to home-based care and BiTE delivery.

Safe BiTE use requires comprehensive risk management across the treatment course. CRS, primarily in the early phase, and, to a lesser extent, ICANS, are key concerns, while infectious complications may persist throughout therapy. Corticosteroids, commonly used to prevent or manage CRS, have been identified as major contributors to infection risk, highlighting the need for alternative management strategies. Anti-IL-6 therapy offers a promising non-immunosuppressive alternative (10). Tocilizumab, an anti-IL-6 receptor (R) monoclonal antibody is widely used for CRS prophylaxis and treatment. In the MajesTEC-1 study, patients who received single dose IV of tocilizumab prior to the first teclistamab step-up dose showed a lower incidence of all-grade CRS compared to the overall study population, 26% vs. 72%, warranting further evaluation of CRS prophylaxis in this setting (2).

Based on our mathematical modeling, siltuximab, a direct IL-6 neutralizing antibody, demonstrates superior control over CRP, a surrogate marker of IL-6 activity (8). Our model defines an intervention threshold at CRP>40 mg/L with a rapid rise over 24 hours. In our cohort, all eight patients who received siltuximab did not develop CRS.

Furthermore, infections were closely linked to corticosteroid use particularly for CRS or ICANS (6). In our small cohort, Infectious complications were limited to three grades <3, events, all bacterial pneumonia, within the first three months. Thus, the use of anti-IL6 strategies may reduce infection risk by minimizing corticosteroid exposure.

Treatment duration was adapted to response, consistent with the approach proposed by van Donk et al. (7), particularly for older patients and currently being investigated explored in ongoing trials. In our series, treatment decisions were guided by a risk-benefit and response assessment. Notably, one patient underwent successful re-treatment following MRD relapse. Additionally, two patients with refractory AL amyloidosis experienced no treatment-related toxicity, achieved complete suppression of serum free light chains, and, in the case of the patient with cardiac involvement, showed a reduction in pro-BNP levels.

These findings support a precision medicine approach integrating immune monitoring and adaptive treatment duration to minimize toxicity while preserving efficacy. This strategy is particularly relevant for elderly or immunocompromised patients, in whom prolonged immunotherapy may further compromise immune function.

The potential superiority of siltuximab over tocilizumab may be attributed to its approximately 100-fold higher affinity for IL-6 and its direct cytokine neutralization, compared to the receptor blockade mechanism of tocilizumab (8). This suggests a dual strategy: tocilizumab may be preferred for prophylaxis (especially when CRP is <40mg/L), given the convenience of sc administration (9) while siltuximab may be more appropriate for therapeutic intervention in patients with elevated CRP levels or grade≥1 CRS.

The feasibility of this strategy in outpatient or home setting depends on robust clinical monitoring. Initial hospital-based administration of IV siltuximab or sc tocilizumab in the initial treatment should be followed by home-based therapy supported by POCT, digital health platforms, and a 24/7 support center. This framework forms the basis of an ongoing prospective study evaluating the clinical, logistical, and economic feasibility of home-based BiTE administration.

## Data Availability

The raw data supporting the conclusions of this article will be made available by the authors, without undue reservation.
